# Heterogeneity of health anxiety in patients following rabies exposure: a latent profile analysis

**DOI:** 10.3389/fpsyg.2026.1818543

**Published:** 2026-04-23

**Authors:** Yue’e Ma, Guodong Xiong, Hua Xie, Lan Liang, Chenghang Mo, Hongbo Li, Xin Liu

**Affiliations:** The Third Affiliated Hospital of Guangxi Medical University, Nanning, China

**Keywords:** health anxiety, health literacy, influencing factors, latent profile analysis, rabies

## Abstract

**Introduction:**

This study aims to identify the various forms of health anxiety in patients exposed to rabies, together with the characteristics and factors influencing the different categories.

**Methods:**

A convenience sampling method was used to recruit patients with rabies exposure treated in the emergency department of a tertiary Grade A hospital in Nanning from January 2024 and April 2025. Data were collected using a general information questionnaire, the Short Health Anxiety Inventory, the Chinese version of the Comprehensive Health Literacy Measurement Scale, and the Perceived Social Support Scale. Latent profile analysis (LPA) was used for identifying and classifying health anxiety in patients. Univariate and unordered multinomial logistic regression analyses were employed to explore the characteristics and factors influencing health anxiety in the various classifications.

**Results:**

Two hundred and twenty-six patients who had been exposed to rabies were enrolled. The LPA results indicated a division of health anxiety into four potential categories, namely, the low-level alleviation (20.30%), low-level fluctuation (31.90%), medium-level fluctuation (32.30%), and high-level maintenance (15.50%) types. Health literacy, perceived social support, educational level, age, medical payment method, and exposure grade were identified as factors influencing these categories of patient health anxiety (*p* < 0.05).

**Conclusion:**

There is heterogeneity in the health anxiety of patients after rabies exposure, suggesting that it is necessary to take a differentiated clinical approach. These findings highlight the importance of stratified psychological interventions and targeted health education programs for patients exposed to rabies, particularly among individuals with limited health literacy and socioeconomic resources.

## 1 Introduction

Rabies, also known as hydrophobia, is an acute zoonotic infectious disease caused by the rabies virus, which primarily invades the central nervous system, due to the lack of specific treatment, the case fatality rate is 100% once symptoms appear (World Health Organization, 2018). Standardized post-exposure prophylaxis is effective in preventing onset of the disease, timely and standardized post-exposure prophylaxis(PEP) is critical to block viral transmission and disease progression (Kessels et al., 2019). However, after exposure, patients commonly experience intense worry regarding the disease prognosis and the effectiveness and safety of the treatment, which can easily lead to significant health anxiety (Lu et al., 2024). Health anxiety(HA) involves excessive health-related worry and fear, typically manifesting in behaviors like persistent reassurance-seeking or avoidance of medical information, It is recognized in the DSM-5 and associated with significant functional impairment, increased healthcare utilization, and poorer health outcomes across diverse populations globally (Scarella et al., 2019). Evidence suggests that significant health anxiety is common following rabies exposure, typically marked by hypervigilance to bodily sensations and a propensity for catastrophic thinking (Warmerdam et al., 2023). This anxiety not only adversely affects treatment compliance, influencing the completion of the standardized post-exposure prophylaxis, but may also lead to unnecessary overutilization of medical services, increasing the burden on both individuals and the healthcare system (Debnath et al., 2025; Dash et al., 2026).

Despite the recognized global burden of HA, research focusing on rabies-exposed individuals remains limited. Most previous studies on rabies exposure have focused on its epidemiological characteristics, standardized management procedures, and evaluation of immunization efficacy, the psychological consequences of exposure have received far less attention (Chen et al., 2026). Existing studies treat HA as a homogeneous construct, overlooking the potential heterogeneity within the patient population, the specific nature and heterogeneity of HA remaining particularly underexplored (Saleem et al., 2020). This limits the development of targeted psychological support. To address this, our study employs Latent Profile Analysis (LPA) to identify distinct subgroups of patients based on their patterns of health anxiety symptoms, rather than treating anxiety as a single trait (Spurk et al., 2020). This study aimed to identify latent profiles of health anxiety among patients exposed to rabies using LPA, and examine demographic, psychosocial, and clinical factors associated with these profiles. The findings are expected to provide an empirical basis for the development of stratified and precise psychological care and health education initiatives, and to offer evidence-based support for the implementation of precise psychological interventions.

## 2 Participant and methods

### 2.1 Participant

A convenience sampling method was used to recruit 226 patients who had been exposed to rabies and had attended the Animal Injury Clinic of a tertiary Grade A hospital in Nanning between January 2024 and April 2025. The inclusion criteria were: (1) Meeting the diagnostic criteria for Grade I-III rabies exposure according to the “Guidelines for Rabies Exposure Prevention and Treatment (2023 Edition)”; (2) Being conscious and having no communication barriers; (3) Aged ≥18 years; (4) Having complete clinical data. The exclusion criteria were: (1) Patients with diagnosed mental or cognitive disorders; (2) Patients with comorbidities such as malignant tumors and end-stage diseases; (3) Participation in other studies. The sample size was determined with reference to methodological guidelines for LPA. LPA, as a finite mixture model, requires an adequate sample to ensure stable and replicable profile solutions. Following recommendations in the methodological literature that a sample size of N ≥ 200 is a prudent minimum when using fit indices like the Bayesian Information Criterion for model selection (Nylund-Gibson and Choi, 2018), we set 200 as our target. This target was then increased by 20% to account for potential attrition and incomplete responses.

### 2.2 Measures

#### 2.2.1 General Information Questionnaire

The General Information Questionnaire was designed by researchers of the current study based on a review of the literature. The questionnaire consisted of two parts: (1) Demographic data: sex, ethnicity, age, marital status, educational level, place of residence (rural, non-rural), employment status (unemployed, retired, employed), monthly household income, medical payment method, and level of knowledge about rabies; (2) Disease-related data: number of comorbid diseases, exposure grade, history of animal injuries, and history of immunization.

#### 2.2.2 Short Health Anxiety Inventory (SHAI)

The Chinese version of the SHAI (CSHAI) revised by Professor Yuan et al. in 2013 was used (Zhang et al., 2015). This scale has good diagnostic accuracy, is effective for studying health anxiety in the Chinese population, and has high internal consistency and reliability. The scale consists of 18 items. The first 14 items pertain to health anxiety, while the last 4 items involve risk factors. It uses a 4-point Likert scale (0 = Never, 1 = sometimes, 2 = often, 3 = always). A score of 15 represents the cutoff point for the presence of anxiety, with higher scores indicating greater health anxiety. The Cronbach’s *α* coefficient is 0.849, indicating good reliability and validity.

#### 2.2.3 Chinese version of the All Aspects of Health Literacy Scale (C-AAHLS)

The Chinese version of the All Aspects of Health Literary Scale (C-AAHLS) translated by Wu et al. was used to assess the level of health literacy in the patients (Wu et al., 2017). The AAHLS contains 11 items across 3 dimensions: ability to use written health information (items 1, 2, 3, 4), ability to communicate with healthcare providers (items 5, 6, 7), and ability to evaluate and apply health information (items 8, 9, 10, 11). Each item has the options of “almost never,” “sometimes,” and “often,” with scoring on a 3-point Likert scale. Items 1 and 4 are reverse-scored, while the other items are positively scored. Higher scores indicate a higher level of health literacy. The scale has good reliability and validity, with an overall Cronbach’s *α* coefficient of 0.811, and Cronbach’s α coefficients for the dimensions ranging from 0.718 to 0.764.

#### 2.2.4 Perceived Social Support Scale (PSSS)

The PSSS was developed by Zimet et al. (1988). The Chinese researchers Jiang Qianjin et al. subsequently translated and modified it into a Chinese version in 2001 according to China’s national conditions. The Chinese version contains 3 dimensions, namely, family support, friend support, and other support, totaling 12 items. It uses a 7-point Likert scale (1 = “strongly disagree,” 7 = “strongly agree”). Higher total scores indicate higher levels of perceived social support. A total score of 61–84 indicates high perceived social support, while scores of 37–60 and 12–36 represent moderate and low perceived social support, respectively. The Cronbach’s *α* coefficient is 0.840 (Huang et al., 2021).

### 2.3 Data collection

The investigator initially screened patients who met the inclusion criteria by reviewing their medical records. After fully explaining the purpose and requirements of the study to the patients and obtaining their informed consent, the investigator used standardized instructions to explain requirements and precautions involved in questionnaire completion. The questionnaires were distributed on-site immediately after the patient’s visit and were completed independently by the patients. Disease-related data, such as the patient’s history of animal injuries and comorbidities, were retrieved from the electronic medical records. Completed questionnaires were checked individually by the investigator item by item. Any omissions were communicated to the patient promptly, and assistance was provided on-site to complete the missing information.

### 2.4 Quality control

Quality supervision was performed during the survey period by several senior nurses in the department who held vaccination certificates. They ensured that investigators used standardized language to explain the survey to patients and checked the completeness of the questionnaires. After the survey, the data were organized by the researcher excluding invalid questionnaires with contradictory selections or patterned responses to ensure the authenticity and reliability of the data. Data entries were double-checked by two individuals to ensure questionnaire accuracy.

### 2.5 Statistical methods

Data were analyzed using SPSS 26.0 and Mplus 8.4 software. The significance level was set at *p* < 0.05. Models with 1 to 5 latent classes were sequentially set for model fit estimation of LPA. Model fit was assessed using the following indices: the Akaike information criterion (AIC), Bayesian information criterion (BIC), adjusted Bayesian information criterion (aBIC), entropy, Lo–Mendell–Rubin adjusted likelihood ratio test (LMRT), and bootstrap likelihood ratio test (BLRT) (Nylund et al., 2007). Lower AIC, BIC, and aBIC values indicate better model fit, while an entropy value closer to 1 indicates more precise classification, and *p* < 0.05 for the LMRT and BLRT suggests that the k-class model fits significantly better than the (k-1)-class model. For comparisons between different health anxiety subtypes, qualitative data were compared using chi-square tests or Fisher’s exact tests, while normally distributed quantitative data are expressed as mean ± standard deviation (x̄ ± s) and were compared using ANOVA, and non-normally distributed quantitative data are expressed as median (interquartile range) (M [P25, P75]) and were compared using the non-parametric Kruskal-Wallis rank sum test. Multinomial logistic regression analysis was used to explore the factors influencing the different health anxiety subtypes, following an assessment of multicollinearity between independent variables.

## 3 Results

### 3.1 LPA results and designation of health anxiety in patients after rabies exposure

LPA was performed on the characteristics of health anxiety in the 226 patients who had been exposed to rabies. The model fit indices are shown in Table 1. As the number of categories increased, the AIC, BIC, and aBIC values tended to decrease. When retaining the 4-class model, the AIC, BIC, and aBIC values were close to their minima, the BLRT *p*-value was < 0.05, and the entropy value was > 0.8, indicating that the fit of this model was superior to that of other models with different numbers of classes. For the 4-class model, the average latent class probabilities for the classes were 20.30, 32.30, 31.90, and 15.50%, respectively.

**Table 1:** Model fit indices for latent profile models of health anxiety characteristics in patients following rabies exposure.

Model	AIC	BIC	aBIC	Entropy	*P*-value		Class probability
					LMR	BLRT	
Model 1	12985.659	13108.798	12994.706	–	–	–	–
Model 2	12562.440	12750.569	12576.261	0.883	<0.001	<0.001	0.301/0.699
Model 3	12404.720	12657.839	12423.315	0.910	<0.001	<0.001	0.203/0.655/0.142
Model 4	12318.817	12636.927	12342.187	0.924	0.021	<0.001	0.203/0.323/0.319/0.155
Model 5	12277.233	12660.333	12305.378	0.929	0.378	<0.001	0.164/0.088/0.394/0.226/0.128

### 3.2 Characteristics and designation of latent classes of health anxiety in patients following rabies exposure

**Figure 1:**
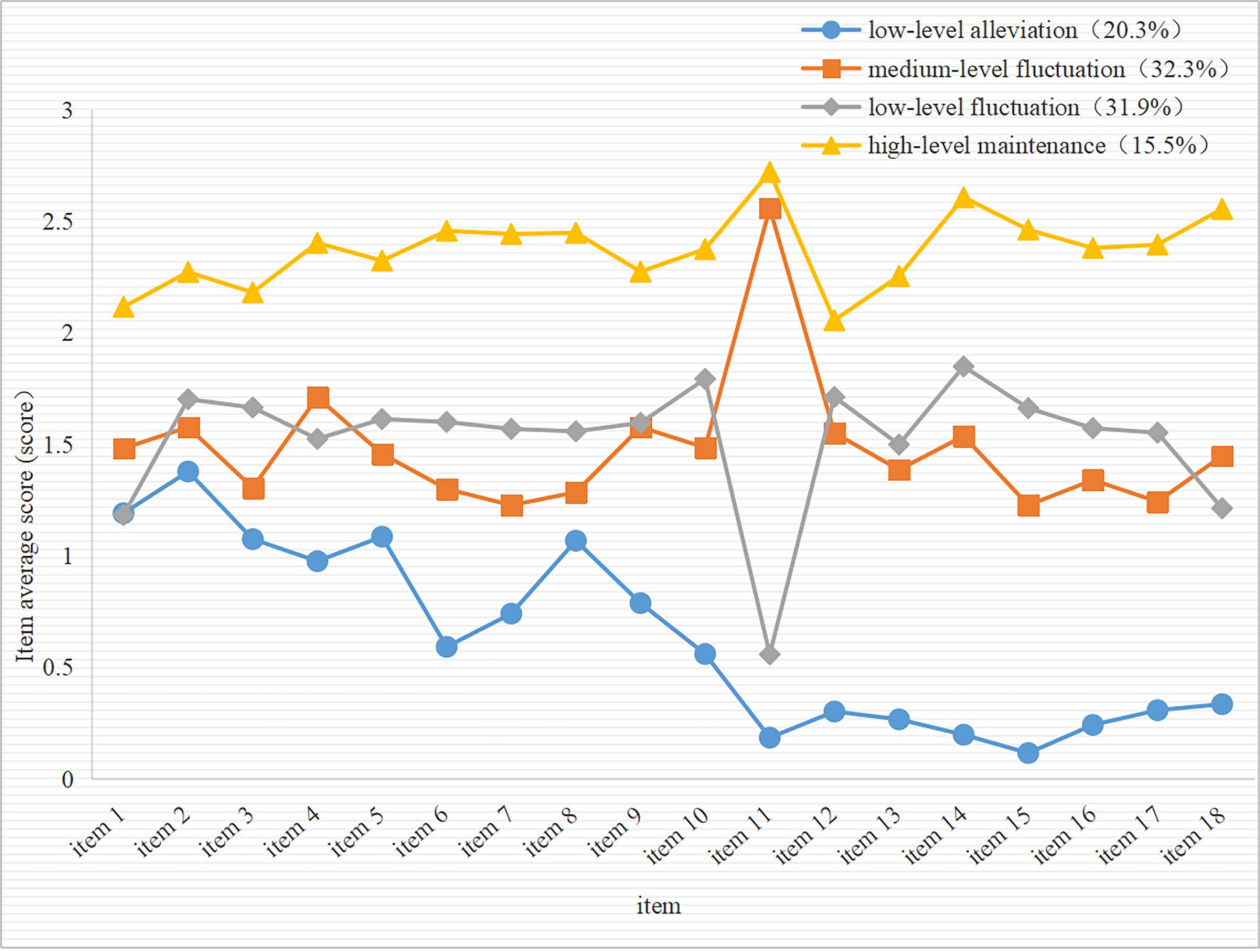
Characteristic distribution of the four latent classes of health anxiety in patients following rabies exposure.

The four latent classes of health anxiety in patients following rabies exposure were designated based on their score patterns across the dimensions of the Health Anxiety Inventory, as shown in Figure 1. Among all classes, Class 1 had the lowest level of health anxiety. Its average item score remained at a low level throughout, with tendency for the anxiety level to reduce; thus, this class was termed the “low-level alleviation type” (20.30%). Class 2 represented a moderate level of health anxiety. Its average item score was significantly higher than that of the low-level group but was lower than that of the high-level group, with obvious fluctuations; it was thus termed the “medium-level fluctuation type” (32.30%). The level of health anxiety in Class 3 was similar to that of the low-level alleviation type on item 1, with both being at a low level, but due to it volatility, it was termed the “low-level fluctuation type” (31.90%). Class 4 represented the highest level of health anxiety among the four classes, with its average item score constant at the highest level throughout, and was thus designated the “high-level maintenance type” (15.50%).

### 3.3 Univariate analysis of the latent profiles of health anxiety in patients following rabies exposure

Differences in demographic characteristics, health literacy scores, and perceived social support scores among the patients in the four health anxiety subtypes were compared. The results showed that the four health anxiety subtypes differed significantly (*p* < 0.05) in terms of age, educational level, medical payment method, level of disease-related knowledge, exposure grade, health literacy score, and perceived social support score. Details are provided in Table 2.

**Table 2:** Univariate analysis of the latent profiles of health anxiety in patients following rabies exposure (*n* = 226).

Variable	Group	Low-level alleviation Type (*n* = 46)	Low-level fluctuation Type (*n* = 72)	Medium-level fluctuation Type (*n* = 73)	High-level maintenance Type (*n* = 35)	Statistic value	*P*-value
Age (years)	–	33.50 ± 14.20	45.10 ± 18.90	39.80 ± 16.50	47.20 ± 20.80	21.35^b^	<0.001
Health literacy score	–	17.20 ± 3.10	11.20 ± 2.00	13.50 ± 2.80	9.00 ± 1.70	125.67^b^	<0.001
Perceived social support score	–	30.10 ± 8.50	45.80 ± 13.90	47.30 ± 11.20	70.50 ± 10.10	98.24^b^	<0.001
Sex [*n* (%)]	Male	24(52.20)	38(52.80)	38(52.10)	18(51.40)	0.03^a^	0.998
	Female	22(47.80)	34(47.20)	35(47.90)	17(48.60)		
Educational level [*n* (%)]	Primary or less	5(10.90)	25(34.70)	22(30.10)	15(42.90)	32.18^a^	<0.001
	Junior High	6(13.00)	22(30.60)	15(20.50)	9(25.70)		
	Senior High	8(17.40)	18(25.00)	19(26.00)	7(20.00)		
	College or above	27(58.70)	7(9.70)	17(23.30)	4(11.40)		
Medical payment method [*n* (%)]	Employee Health Insurance	18(39.10)	12(16.70)	15(20.50)	4(11.40)	30.75^a^	<0.001
	Resident Health Insurance	12(26.10)	20(27.80)	19(26.00)	7(20.00)		
	Out-of-pocket	16(34.80)	40(55.60)	39(53.40)	24(68.60)		
Disease knowledge level [*n* (%)]	No knowledge	10(21.70)	40(55.60)	35(47.90)	22(62.90)	38.92^a^	<0.001
	Partial knowledge	18(39.10)	25(34.70)	30(41.10)	10(28.60)		
	Full knowledge	18(39.10)	7(9.70)	8(11.00)	3(8.60)		
Exposure grade [*n* (%)]	Grade I	15(32.60)	8(11.10)	12(16.40)	1(2.90)	28.44^a^	<0.001
	Grade II	16(34.80)	24(33.30)	25(34.20)	12(34.30)		
	Grade III	15(32.60)	40(55.60)	36(49.30)	22(62.90)		
History of Animal Injury [*n* (%)]	Yes	25(54.30)	48(66.70)	45(61.60)	20(57.10)	2.48^a^	0.479
	No	21(45.70)	24(33.30)	28(38.40)	15(42.90)		

a χ^2^ value.

b F-value.

### 3.4 Multivariate analysis of the latent profiles of health anxiety in patients following rabies exposure

Using the latent health anxiety classification as the dependent variable and variables that showed statistical significance in the univariate analysis as independent variables, multinomial logistic regression analysis was performed using the low-level alleviation type as the reference group. The independent variables were assigned as follows: Educational level: primary school or below = 1, junior high school = 2, senior high school = 3, college or above = 4; Level of disease-related knowledge: no knowledge = 1, partial knowledge = 2, full knowledge = 3; Medical Payment Method: employee health insurance = 1, resident health insurance = 2, out-of-pocket = 3; Exposure Grade: grade I = 1, grade II = 2, grade III = 3. Measurement data were entered as their original values. The results showed that health literacy, perceived social support, educational level, age, medical payment method, and exposure grade were factors influencing health anxiety classification in patients following rabies exposure (*P* < 0.05). Compared to the Low-Level Alleviation Type, the significant predictors for belonging to the Medium-Level Fluctuation Type included: lower health literacy [OR (95%CI):0.49(0.36, 0.67)], higher perceived social support [OR (95%CI):1.09(1.03, 1.16)], and lower educational attainment, with the highest risk observed for those with primary education or below [OR (95%CI):17.30(3.97, 75.38)]. The significant predictors for belonging to the High-Level Maintenance Type included: lower health literacy [OR (95%CI):0.26(0.17, 0.40)], higher perceived social support [OR (95%CI):1.20(1.11, 1.30)], and lower educational attainment, with the highest risk observed for those with primary education or below [OR (95%CI):14.88(2.76, 80.17)]. Additionally, older age [OR (95%CI):1.07(1.03, 1.11)] and out-of-pocket medical payment [OR (95%CI):7.03(2.05, 24.11)] significantly increased the odds of belonging to this profile. For the Low-Level Fluctuation Type, significant factors included lower health literacy [OR (95%CI):0.36(0.25, 0.52)], older age [OR (95%CI):1.06(1.02, 1.10)], out-of-pocket payment [OR (95%CI):4.26(1.63, 11.14)], and Grade III exposure [OR (95%CI):5.00(1.70, 14.71)]. Details are shown in Table 3.

**Table 3:** Multinomial logistic regression analysis of latent classes of health anxiety in patients following rabies exposure (*n* = 226).

Subtype	Item		*β*	*SE*	Wald χ^2^	*P*-value	*OR* (95%*CI*)
Medium-level fluctuation type	Health literacy score	–	−0.71	0.16	19.70	<0.001	0.49(0.36, 0.67)
	Perceived social support score	–	0.09	0.03	9.00	0.003	1.09(1.03, 1.16)
	Educational level	Primary or below	2.85	0.75	14.44	<0.001	17.30(3.97, 75.38)
		Junior High	1.92	0.68	7.97	0.005	6.82(1.80, 25.87)
		Senior High	1.58	0.65	5.91	0.015	4.85(1.36, 17.30)
Low-level fluctuation type	Health literacy score	–	−1.02	0.18	32.11	<0.001	0.36(0.25, 0.52)
	Age	–	0.06	0.02	9.00	0.003	1.06(1.02, 1.10)
	Medical payment method	Out-of-pocket	1.45	0.49	8.76	0.003	4.26(1.63, 11.14)
	Exposure grade	Grade III	1.61	0.55	8.56	0.003	5.00(1.70, 14.71)
High-level maintenance type	Health literacy score	–	−1.35	0.22	37.69	<0.001	0.26(0.17, 0.40)
	Perceived social support score	-	0.18	0.04	20.25	<0.001	1.20(1.11, 1.30)
	Age	-	0.07	0.02	12.25	<0.001	1.07(1.03, 1.11)
	Educational level	Primary or below	2.70	0.86	9.86	0.002	14.88(2.76, 80.17)
	Medical payment method	Out-of-pocket	1.95	0.63	9.60	0.002	7.03(2.05, 24.11)

The low-level alleviation type was used as the reference group for all comparisons.

## 4 Discussion

### 4.1 Heterogeneity of health anxiety in patients following rabies exposure

In this study, four distinct health anxiety spectra were identified: low-level remission type (20.30%), low-level fluctuation type (31.90%), medium-level fluctuation type (32.30%), and high-level maintenance type (15.50%). This classification indicates significant heterogeneity in health anxiety within this population, challenging the assumption of group homogeneity that is often implicit in many previous variable-focused studies of psychological distress following health threats. Notably, nearly half (47.80%) of the patients exhibited moderate to severe anxiety, highlighting that severe psychological distress is a prevalent comorbidity that necessitates clinical attention and standard post-exposure prophylaxis (Asmundson et al., 2010). The observed heterogeneity aligns with emerging literature on psychological responses to other acute health threats, such as pandemic viruses or cancer, where person-centered analyses have similarly identified distinct subgroups with varying needs (Shevlin et al., 2020; Stanton et al., 2018). The mechanisms underlying this heterogeneity may involve the interaction of individual factors, such as differences in cognitive evaluation, coping resources, and pre-existing vulnerabilities with stress exposure events, leading to divergent anxiety trajectories (Carver and Smith, 2010). These findings carry direct clinical implications, suggesting the incorporation of brief routine psychological assessments into standard post-exposure management to identify high-risk patients for targeted support. At the policy level, this evidence advocates for the development of structured, stratified treatment pathways within rabies prevention programs, ensuring that mental health support is recognized as a core component of comprehensive post-exposure care, alongside wound management and immunization (World Health Organization, 2018; Ding et al., 2025).

### 4.2 Factors influencing health anxiety in patients following rabies exposure

#### 4.2.1 Health literacy

Health literacy emerged as the most consistent protective factor, while low levels of relief type significantly increased the likelihood of belonging to all three higher anxiety types compared to low levels of relief type. The graded protective effect diminished from 0.49 to 0.26 with increasing anxiety severity, suggesting a stronger protective effect against more severe manifestations of anxiety. This finding is consistent with previous research on the effect of health literacy on psychological outcomes, higher health literacy is associated with improved mental health outcomes across various conditions, including reduced anxiety and depression in individuals with chronic conditions (Magallón-Botaya et al., 2023; Haeri-Mehrizi et al., 2024). The graded protective effect observed in our study is consistent with patterns identified in studies examining health literacy and health outcomes, where enhanced literacy skills confer additional advantages in more complex health scenarios (Li, 2022). The protective mechanism may involve individuals with higher health literacy mitigating catastrophic misconceptions about symptoms and feelings of helplessness by comprehending the transmission of rabies and the efficacy of PEP (Kikas et al., 2024; Asmundson et al., 2010). Furthermore, high health literacy enables them to communicate more effectively with medical staff and convey information more clearly (Murugesu et al., 2022; Wernick et al., 2016). It is essential to integrate health literacy into rabies prevention communications and ensure that all public health messages regarding rabies are accessible to individuals across all literacy levels.

#### 4.2.2 Perceived social support

Social support is usually regarded as a protective psychological resource; however, this study reveals that patients who experience high levels of maintenance anxiety actually receive the highest degree of support. This finding is consistent with recent research results in the fields of health anxiety and somatoform disorders (Liu et al., 2024; Mazhari et al., 2022). Excessive worry and repeated reassurance from family members may unintentionally reinforce beliefs and anxiety related to the disease rather than promoting effective emotional regulation, which is in line with previous studies (Rector et al., 2011). This finding has direct clinical significance, emphasizing the need to expand the scope of intervention measures to not only include the patient themselves but also their close social network. Family members should be guided to provide positive and constructive support.

#### 4.2.3 Socioeconomic factors

The significant influence of socioeconomic factors, such as out-of-pocket medical payments and low educational levels, cannot be overlooked. Economic pressures directly translate into concerns about treatment costs, thereby exacerbating the psychological burden on patients. This is evidenced by the well-documented concept of financial toxicity in oncology, where high out-of-pocket costs are strongly associated with treatment-related distress, anxiety, and diminished quality of life (Lentz et al., 2019). This phenomenon extends beyond chronic illness, as research in emergency care confirms that perceived financial burden following acute medical events is a significant, independent predictor of poorer psychological outcomes, including post-traumatic stress symptoms (Ioannou et al., 2018). Furthermore, lower educational attainment often limits an individual’s health literacy—their ability to acquire, comprehend, and utilize health-related information. This renders them more vulnerable to misunderstandings and fear during health threats, a pathway supported by studies linking limited health literacy to worse psychological adjustment in chronic conditions like diabetes (Stormacq et al., 2019). The COVID-19 pandemic further highlighted this disparity, demonstrating that populations with lower education levels faced greater challenges in processing complex public health information, leading to heightened confusion and anxiety (Paakkari and Okan, 2020). Collectively, this evidence underscores that health anxiety, particularly in contexts like rabies exposure, is not merely an individual psychological issue but a psychosocial problem deeply rooted in and exacerbated by structural social inequalities (Braveman et al., 2011). Consequently, macro-level policy interventions that address these root causes are essential. Effective measures could include enhancing health insurance coverage for post-exposure prophylaxis to directly alleviate the financial shock associated with treatment (Powell et al., 2016), alongside the development and dissemination of targeted, easy-to-understand health education materials designed with universal health literacy principles for lower-education populations (Mabachi et al., 2016). Such strategies constitute a form of foundational social support aimed at mitigating the psychological burden at its socioeconomic source.

#### 4.2.4 Risk associated with age and exposure grade

Advanced age was identified as a common risk factor for both the low-level fluctuation and high-level maintenance types of health anxiety. Studies indicate that, compared to younger patients, older patients exposed to rabies are more likely to suffer from health anxiety, which tends to be persistent and recurrent. This phenomenon may be related to factors such as age-related changes in cognitive function, a generally heightened fear of death and disease, and a higher prevalence of comorbid chronic conditions among the elderly. These factors collectively render them more sensitive and vulnerable to the threat of death associated with rabies (El-Gabalawy et al., 2013). Grade III exposure was identified as a specific risk factor for the low-level fluctuation type of health anxiety. This direct dose–response relationship between physical trauma severity and psychological impact is well established in trauma research, as seen in studies of accident victims where injury severity correlates with subsequent anxiety levels (Wu and Cheung, 2006). This finding reflects the direct association between the severity of exposure and the degree of psychological trauma. More severe wounds and exposure history are associated with more intense initial fear, potentially triggering and sustaining the state of anxiety (Forkus et al., 2023).

### 4.3 Implications for precision interventional strategies

By analyzing the multiple factors influencing the different classes of health anxiety, this study provides precise empirical evidence for the construction of a comprehensive interventional system integrating psychological support, health education, guidance on family support, and socioeconomic assistance. For patients with medium-level fluctuation-type anxiety, efforts should focus on increasing health literacy, providing targeted education on rabies, particularly in groups with low educational levels, and communicating in intuitive and easy-to-understand way (Magallón-Botaya et al., 2023). For patients with low-level fluctuation-type anxiety, attention should be paid to older patients and those facing financial difficulties (out-of-pocket payment). Interventions should include clear explanations of cost, information on channels providing financial assistance, and increased psychological support tailored for this population. The high-level maintenance type is associated with the greatest need for professional psychological intervention. Apart from education in health literacy, it is crucial to identify and address their pathological social-support patterns by providing joint counseling for both patients and their families (Taylor et al., 2004). Furthermore, assessment for comorbid hypochondriasis should be considered, with potential referral to psychiatric or psychological counseling services for specialized interventions such as cognitive behavioral therapy (Veddegjærde, et al., 2020).

## 5 Limitations

There were some limitations in this study. First, the cross-sectional design limits causal inference between the identified factors and health anxiety profiles. Second, the reliance on self-reported questionnaires may introduce response bias. Third, the single-center sampling strategy may limit the generalizability of the findings. Future multicenter longitudinal studies are needed to validate the stability of these latent profiles over time.

## 6 Conclusion

This study demonstrates significant heterogeneity in health anxiety among patients exposed to rabies. Health literacy, perceived social support, socioeconomic conditions, and exposure severity were important determinants of anxiety profiles. These findings highlight the need for integrating psychological assessment and targeted educational interventions into rabies post-exposure management programs.

## Data Availability

The original contributions presented in the study are included in the article/supplementary material, further inquiries can be directed to the corresponding author.
